# Digital NFATc2 Activation per Cell Transforms Graded T Cell Receptor Activation into an All-or-None IL-2 Expression

**DOI:** 10.1371/journal.pone.0000935

**Published:** 2007-09-26

**Authors:** Miriam Podtschaske, Uwe Benary, Sandra Zwinger, Thomas Höfer, Andreas Radbruch, Ria Baumgrass

**Affiliations:** 1 German Rheumatism Research Centre, Berlin, Germany; 2 Institute of Medical Immunology, Charité, Humboldt-University Berlin, Berlin, Germany; 3 Department of Theoretical Biophysics, Humboldt-University Berlin, Berlin, Germany; Institut de Biochimie et Biophysique Moléculaire et Cellulaire, CNRS/UMR 8619, University Paris Sud, France

## Abstract

The expression of interleukin-2 (IL-2) is a key event in T helper (Th) lymphocyte activation, controlling both, the expansion and differentiation of effector Th cells as well as the activation of regulatory T cells. We demonstrate that the strength of TCR stimulation is translated into the frequency of memory Th cells expressing IL-2 but not into the amount of IL-2 per cell. This molecular switch decision for IL-2 expression per cell is located downstream of the cytosolic Ca^2+^ level. Here we show that in a single activated Th cell, NFATc2 activation is digital but NF-κB activation is graded after graded T cell receptor (TCR) signaling. Subsequently, NFATc2 translocates into the nucleus in an all-or-none fashion per cell, transforming the strength of TCR-stimulation into the number of nuclei positive for NFATc2 and IL-2 transcription. Thus, the described NFATc2 switch regulates the number of Th cells actively participating in an immune response.

## Introduction

One of the most relevant consequences of the activation of T lymphocytes through antigen is the de novo synthesis of interleukin (IL)-2. This cytokine is a key molecule in TCR dependent activation of Th lymphocytes, affecting proliferation, differentiation, apoptosis, and tolerance [1]–[3]. IL-2 also plays an essential and non-redundant role in the control of peripheral T cell tolerance by regulatory T cells (reviewed in [4]–[6]). Regulatory T cells require IL-2 for their generation, maintenance and function [7]; [8]. The multiple auto- and paracrine functions of IL-2 raise the question about the accurate execution of the IL-2 gene expression program which is essential for the function of the adaptive immune system and T cell homeostasis.

In this study, we show that in human Th lymphocytes, IL-2 expression at the level of an individual T cell is regulated as an all-or-none decision. This translation of a graded TCR signal into the observed binary decision process requires a molecular switch in the signaling cascade regulating the expression of IL-2. Elements controlling IL-2 expression include the combined activation of TCR and co-stimulatory receptor [9], a certain threshold level of cytosolic Ca^2+^ [10]; [11], the cooperative action of nuclear factors of activated T cells (NFAT), NF-κB and AP-1 [12]–[14], chromatin remodeling [15]; [16], transcription [16], message stability [17], and translation [18]. For a binary IL-2 expression Fiering et al. [19] and Piron and Elston [20] have suggested that the thresholds for IL-2 expression in individual cells are set by the concentrations of transcriptionally active NFAT and NF-κB, as well as the cooperation between them, with a stochastic DNA binding of those transcription factors due to limited availability.

Here, we show that the expression of IL-2 in individual human Th lymphocytes is binary, follows an all-or-none rule and is controlled by NFATc2 as molecular switch. NFATc2 is cooperatively and quantitatively dephosphorylated by calcineurin in individual Th cells that are activated above a threshold following TCR stimulation. The entire contingent of NFATc2 within an individual cell consequently translocates into the nucleus and induces the expression of IL-2. As a molecular switch, NFATc2 also controls the expression of other genes in activated Th cells, shown here for interferon-γ (IFN−γ). Therefore, NFATc2 is a general checkpoint, controlling the participation of activated Th cells in an immune reaction in a binary fashion.

## Results

### Binary expression of IL-2

**Figure 1:**
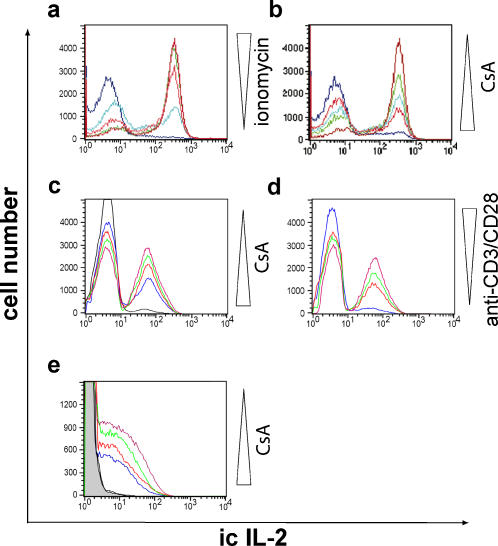
Binary expression pattern of IL-2 in human Th cells. Histogram overlay of IL-2 expression in peripheral human CD4^+^ T cells after stimulation with PMA and ionomycin for 5 h, anti-CD3/CD28 antibodies or peptide-loaded APC for 14 h. IL-2 expression was detected by intracellular (ic) staining. (a) Constant PMA (10 ng/ml) and ionomycin concentrations ranging from 100 to 1000 ng/ml were used. The color codes for ionomycin concentrations are: 100 ng/ml dark blue; 200 ng/ml light blue; 300 ng/ml red; 500 ng/ml green; 1 µg/ml dark red. (b) Constant PMA (10 ng/ml) and ionomycin concentrations (1000 ng/ml) were used in the presence of 2–10 nM CsA. The color codes for CsA concentrations are: no CsA dark red; 2 nM green; 3 nM light blue; 5 nM red; 10 nM dark blue. (c) Anti-CD3/CD28 antibodies coated Facs-tubes were used for stimulation of memory T cells in presence of 2–50 nM CsA. The color codes for CsA concentrations are: no CsA dark red; 2 nM green; 5 nM red; 10 nM blue; 50 nM black. (d) Facs tubes were coated with the following concentrations of anti-CD3/CD28 antibodies (ratio 1∶4): 0.075 and 0.3 µg/ml dark blue; 0.15 and 0.6 µg/ml red; 0.3 and 1.2 µg/ml green; 0.5 and 2.0 µg/ml dark red. (e) CMV-peptide-loaded APC were used in the presence of 2–50 nM CsA. The color codes for CsA concentrations are: no CsA dark red; 2 nM green; 5 nM red; 10 nM dark blue; 50 nM black. APC loaded with an unspecific CMV-peptide were used as a stimulation control (grey-filled). Results are representative for at least two independent experiments.

In these experiments, we showed that, in human CD4^+^ cells, a modulated Ca^2+^ influx induced by different ionomycin concentrations changed the amount of IL-2-expressing cells rather than the intensity of IL-2 expression per cell. Thus, the strength of simulated TCR signaling correlates to the proportion of T cells that produce IL-2 at the level of a population of CD4^+^ cells. However, different ionomycin concentrations did not change the mean fluorescence intensities (MFI) of IL-2, indicating a binary decision for IL-2 expression at the level of an individual T cell (**Fig. 1a**).

One major translator of Ca^2+^-signals into T cell activation is the Ser/Thr protein phosphatase calcineurin, which is activated by Ca^2+^ and calmodulin. We examined the effect of the classical calcineurin inhibitor cyclosporin A (CsA) on IL-2 expression in stimulated T cells. In agreement with the data from our experiments with different ionomycin concentrations, treatment of T cells with increasing concentrations of CsA during stimulation with high amounts of PMA/ionomycin (10 ng/ml and 1 µg/ml) influenced the frequency of IL-2 producing cells but not the intensity of the IL-2 production per cell. This was shown by both, flow cytometric (**Fig. 1b**) and ELISPOT analysis **(Supplementary Fig. S1)**. Using the direct activation of surface receptors with anti-CD3/CD28 antibodies and different concentrations of CsA (**Fig. 1c**) or different amounts of antibodies (**Fig. 1d**), we confirmed that IL-2 production is a binary decision process under conditions more closely resembling the antigen specific stimulation of T cells. The stimulation of a human cytomegalovirus (CMV) specific T-cell line with CMV peptide-pulsed autologous APC in the presence of different CsA concentrations confirmed the binary IL-2 expression in antigen-stimulated T cells (**Fig. 1e**).

### The binary decision for expression of IL-2 occurs upstream of il-2 transcription

**Figure 2:**
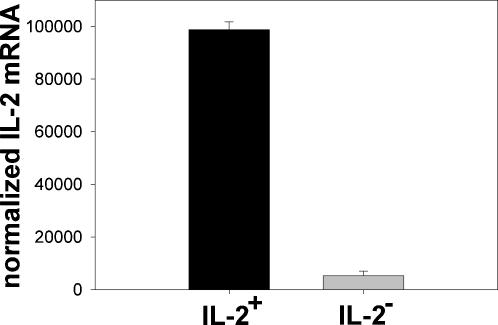
Sorted IL-2 secreting and non-secreting Th cells differ in their IL-2 mRNA level. Human Th cells were sorted for IL-2 secretion 2 h after activation with PMA/ionomycin. The IL-2 mRNA levels were analyzed in IL-2 secreting and non-secreting cell populations using qRT-PCR. The results were normalized to unstimulated control cells. One representative experiment with three parallel samples is shown.

For the molecular analysis of cells expressing IL-2 or not, we separated IL-2 secreting and non-secreting cells from cultures of ex vivo isolated CD4^+^ lymphocytes, which had been stimulated with PMA/ionomycin for 2 hours, using an IL-2 capture assay (Miltenyi Biotec, Bergisch Gladbach, Germany) [21]; [22]. Re-analysis of the sorted fractions confirmed the successful separation of cells expressing IL-2 from cells not expressing IL-2, with a purity of>98% for both the IL-2 secreting and non-secreting cell populations. The isolated IL-2 secreting cells had a 25-fold greater IL-2 mRNA-content than the cells not secreting IL-2 (**Fig. 2**), indicating that the expression of IL-2 in the activated Th cells is regulated upstream of transcription.

### NFATc2 versus NF-κB translocation into the nucleus

Both NF-κB and NFATc2 link calcineurin with il-2 transcription [9]; [23]. To analyze the availability of NF-κB and NFATc2 in the nuclei of activated Th cells, we developed a method to detect isolated nuclei stained with NF-κB- and NFATc2-specific fluorescent antibodies by flow cytometry (**Supplementary Fig. S2**). Nuclei were gated according to forward and side scatter and propidium iodide staining and excluded from doublets by pulse-processing (**Supplementary Fig. S3**). The specificity of staining for NFATc2 and NF-κB (p65) was confirmed using nuclei from cells pre-treated with specific inhibitors of calcineurin for NFATc2 and nuclei from cells stimulated with TNF-α (an alternative NF-κB inducer) for NF-κB (**Supplementary Fig. S4**).

**Figure 3:**
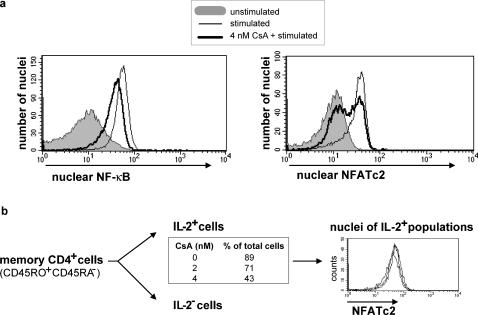
Nuclear translocation of NFATc2 but not NF-κB is binary. (a) The amounts of NF-κB (p65) and NFATc2 were measured in isolated nuclei of memory Th cells (CD4^+^CD45RO^+^CD45RA^-^) using flow cytometry. Before preparation of the nuclei, cells were stimulated with PMA/ionomycin or left unstimulated in the presence or absence of 4 nM CsA for 2 h. One representative experiment out of three is shown. (b) Nuclei of sorted IL2 secreting cells were stained for NFATc2 after pre-incubation of memory Th cells (CD4^+^CD45RO^+^CD45RA^-^) with either DMSO (control), 2 nM or 4 nM CsA and subsequent stimulation with PMA/ionomycin for 2 h. The diagram shows a similar MFI in the histogram overlay of the three samples for one representative experiment out of two.

Human memory Th cells (CD4^+^, CD45RO^+^, CD45RA^-^) were stimulated with PMA/ionomycin in the presence or absence of 4 nM CsA (IC_50_). In the absence of CsA, 70–92% of the activated Th cells expressed IL-2 after stimulation, with relatively large inter-individual variations. The presence of 4 nM CsA reduced the number of IL-2 expressing cells by approximately half to 30–50%. In the experiment shown in **Figure 3a**, 87% of the cells expressed IL-2 and all of the nuclei of these cells stained completely positive for NF-κB and NFATc2. In the presence of CsA, only 49% of the activated cells expressed IL-2. However, all nuclei were stained for NF-κB, though less intense, with a difference in mean fluorescence intensity (ΔMFI) of 31% (**Fig. 3a**, left). In contrast, NFATc2 staining correlated with IL-2 expression in CsA treated cells in the fact that 57% of the nuclei were positively stained for NFATc2 (MFI = 36.7) with the same MFI as the nuclei stimulated in the absence of CsA (MFI = 34.1) (**Fig. 3a**, right). The nuclei which did not stain for NFATc2 (MFI = 11.9) had the same MFI as the nuclei from unstimulated Th cells (MFI = 11.2). This result shows that, in response to graded TCR signaling, NF-κB translocates into the nucleus in a graded fashion, while the translocation of NFATc2 into the nucleus occurs in a binary fashion. This qualifies NFATc2 or upstream molecules as a molecular switch for the binary control of IL-2 expression. In fact, in IL-2 secreting cells isolated from memory Th cells activated with PMA/ionomycin and partially inhibited by 2 or 4 nM CsA, all nuclei contained NFATc2 and all stained with the same MFI (**Fig. 3b**, right), confirming that the quantitative translocation of NFATc2 into the nucleus is a hallmark of IL-2 expressing Th cells after TCR stimulation.

### Binary NFATc2 nuclear translocation is regulated by calcineurin

The graded inhibition of calcineurin by 2 nM CsA and 4 nM CsA had increased the fraction of IL-2 non-producing cells in the experiment shown in **Figure 3b** from 11% (control) to 29% and 57%, respectively. However, the graded inhibition of calcineurin resulted in a binary decision, sending NFATc2 to the nucleus quantitatively, but in a decreasing fraction of cells (numbers are given in **Fig. 3b**).

**Figure 4:**
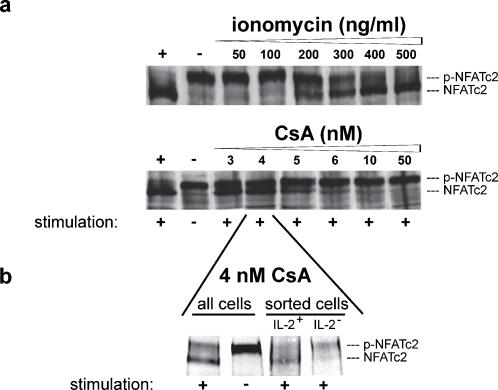
Either all-or-no NFATc2 is dephosphorylated per cell. (a) Immunodetection of phosphorylated and dephosphorylated NFATc2 after activation of human memory Th cells (CD4^+^CD45RO^+^CD45RA^-^) for 1 h with either constant concentration of PMA (10 ng/ml) and increasing concentration of ionomycin (50–1000 ng/ml) or PMA/ionomycin (10 ng/ml and 1000 ng/ml) in presence of the depicted CsA concentrations (3–50 nM). (b) Immunodetection of NFATc2 in cell populations after PMA/ionomycin stimulation in the presence of 4 nM CsA for 2 h and subsequent sorting for IL-2 secreting and non-secreting cells. Unsorted cells served as controls.

The translocation of NFATc2 into the nucleus is controlled by calcineurin through dephosphorylation in 13 phospho-serines of NFATc2 [24]. In accordance with previous work, we found that in cells subjected to a graded stimulation with ionomycin (50–500 ng/ml, in the presence of 10 ng/ml PMA), or a graded inhibition of stimulation with PMA (10 ng/ml) and ionomycin (1 µg/ml) by CsA (2–50 nM), there is a variation in the ratio of completely phosphorylated versus completely dephosphorylated NFATc2, with no detectable partially dephosphorylated NFATc2 (**Fig. 4a**). The ratio of dephosphorylated versus phosphorylated NFATc2 correlates with the proportions of IL-2 producing versus non-producing cells (**Fig. 1b**). By separation of IL-2 secreting versus non-secreting cells after stimulation in presence of 4 nM CsA we dissected that this correlation is due to an all-or-none NFATc2 dephosphorylation per cell. Integrated fluorescence intensity of both NFATc2 bands showed a stronger NFATc2 dephosphorylation in the IL-2 positive fraction (66.5%) than in the IL-2 negative fraction (37.6%).

### Binary expression of IFN-γ but not CD69

**Figure 5:**
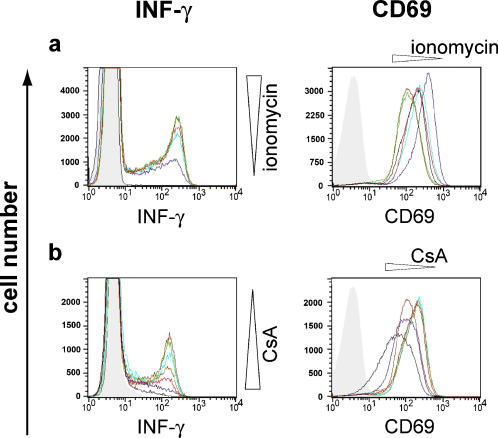
IFN-γ shows a binary expression pattern in partially stimulated human memory CD4^+^ T cells, while CD69 displays a graded expression pattern in these cells. Histogram overlay of protein expression in memory Th cells (CD4^+^CD45RO^+^RA^-^) after stimulation with PMA and ionomycin for 5 h. IFN-γ expression was detected by intracellular (ic) and CD69 by surface staining. (a) Constant PMA (10 ng/ml) and ionomycin concentrations ranging from 100 to 1000 ng/ml were used. The color codes for ionomycin concentrations are: no ionomycin black; 100 ng/ml dark blue; 200 ng/ml light blue; 300 ng/ml red; 500 ng/ml green; 1000 ng/ml dark red. (b) Constant PMA (10 ng/ml) and ionomycin concentrations (1000 ng/ml) were used in the presence of 2–50 nM CsA. The color codes for CsA concentrations are: no CsA dark red; 2 nM green; 3 nM light blue; 4 nM brown; 5 nM red; 10 nM dark blue; 50 nM black. The unstimulated control samples are shown as light grey areas. One representative experiment out of two is shown.

Expression of IFN-γ, another NFAT-dependent gene [25], was found to be as binary as IL-2 expression under conditions of partial activation both with smaller amounts of ionomycin and with inhibition of PMA/ionomycin or anti-CD3/CD28 stimulation by CsA (**Fig. 5**, data not shown). Co-staining of IL-2 and IFN-γ in memory Th cells (CD4^+^, CD45RO^+^, CD45RA^-^) showed that the majority of IFN-γ producing cells co-express IL-2 and that IFN-γ and IL-2 expression are strictly correlated to each other (**Supplementary Fig. S5**). In the experiment shown, only 2–3% of the memory cells produce IFN-γ without IL-2. Stimulation with PMA/ionomycin and anti-CD3/CD28 results in fewer IFN-γ producing cells (one representative experiment: 23%, 13%) than IL-2 producing cells (one representative experiment: 73%, 45%). However, the strength of simulated TCR signaling correlates to the proportion of T cells that produce IFN-γ and did not change the MFI and, therefore, the amount of IFN-γ per cell (**Fig. 5**).

After graded induction or inhibition of the TCR signaling pathway, we observed a graded expression of the NFAT-independent gene CD69 [26]; [27] (**Fig. 5**). In contrast to IL-2 and IFN-γ, CD69 expression is induced by PMA alone. Despite a unidirectional effect of both ionomycin (100–1000 µg/ml) and CsA (2–50 nM) on CD69 expression, PMA alone induced MFI-values of CD69 between those found in the other groups (**Fig. 5**). This effect may be caused by different combinations and strengths of various pathways influencing each other [26].

### Mathematical modeling of binary cytokine expression by cooperative activation of NFATc2

**Figure 6:**
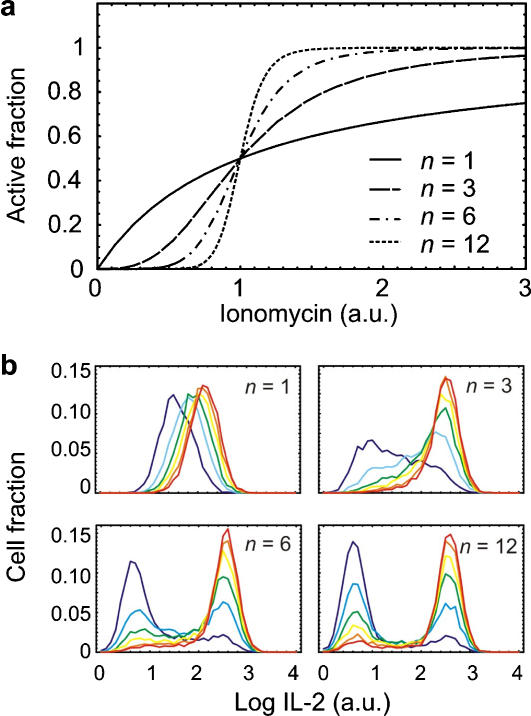
Mathematical modeling of cytokine expression. (a) Response curves of transcription factor activity versus activating signal (ionomycin), are shown modeled as a Hill function (see Methods). Regulation of NFAT activity by a single phosphorylation site would result in a hyperbolic response (Hill coefficient n = 1), while cooperative dephosphorylation of multiple sites would give rise to sigmoid responses (n>1). (b) Simulated patterns of cytokine expression. The ionomycin stimulus increases from blue to red in steps of 0.125, running from 0.125 to 0.75 for n = 1, and steps of 0.5, running from 0.5 to 3.0 for n>1. With increasing steepness of the response curve, cytokine induction changes from gradual to binary.

We mathematically modeled the expression of an NFATc2-dependent gene. NFATc2 could exhibit different responses to an ionomycin stimulus, depending on the degree of cooperativity in the dephosphorylation of NFATc2. The stimulus-response curves can be fitted by a Hill function, where the Hill coefficient *n* is a measure for their steepness. Generally, the Hill coefficient increases with the number of serine residues whose dephosphorylation contributes to the activation of NFATc2 [28]. If NFATc2 would be activated through dephosphorylation of a single serine residue (*n* = 1), a hyperbolic response curve for the nuclear localization of NFATc2 would result. In contrast, the cooperative dephosphorylation of multiple serine residues would give rise to sigmoid response curves, the steepness of which increased with the number of serines involved (*n* = 3–12) [28] (**Fig. 6a**). We simulated the resulting gene expression for graded stimulation with ionomycin, assuming that the levels of active calcineurin and total NFATc2 display random variations in a cell population (**Fig. 6b**). For non-cooperative NFATc2 activation (n = 1), the expression pattern is graded, whereas for high cooperativity (*n* = 12), the pattern is binary. The transition from graded to binary expression occurs in an incremental fashion with increasing cooperativity. First, responses become more mixed but retain their graded component (*n* = 3). For *n* = 6 and larger, the binary pattern is fully developed and closely matches the experimentally observed pattern for IL-2 (**Fig. 1b** and **1c**). A response curve with the Hill coefficient *n* = 6 would result if 7 [28] out of the 13 conserved serines [24] contribute to a cooperative conformational switch that enables NFATc2 activation and nuclear translocation.

## Discussion

In this study, we have verified that the transcriptional response of IL-2 is not a graded but a binary decision process in stimulated Th cells. This is documented by several results. First, both anti-CD3/CD28 and PMA/ionomycin stimulation clearly showed two distinct populations: IL-2 producing cells and non-producing cells. However, both stimulating agents differ in their potential to activate T cells and, therefore, induced different numbers of IL-2 producing cells. The stimulation of a human cytomegalovirus (CMV) specific T-cell line with CMV peptide-pulsed autologous APC also induced IL-2 expression but did not show two seperated populations. Second, titration of ionomycin in the presence of invariably large amounts of PMA only changed the frequency of IL-2 producing cells but not the intensity of IL-2 expression per cell in all T cell populations examined: Th cells (CD4^+^), naïve Th cells (CD4^+^CD45RA^+^CD45RB^-^), and memory Th cells (CD4^+^CD45RA^-^CD45RB^+^) (**Fig. 1a** and data not shown). Third, the concentration of the classical calcineurin inhibitor CsA showed an inverse correlation to the number of IL-2 producing cells but not to the amount of IL-2 per cell. As with ionomycin titration, the mean fluorescence intensity of the population of IL-2 producing cells did not change by CsA titration. This was true for all three stimuli used: PMA/ionomycin, anti-CD3/CD28 antibodies and CMV peptide-pulsed autologous APC. Forth, by measurement of IL-2 secretion using ELISPOT analysis, we confirmed our results from flow cytometric analysis.

In consequence, the question arises at which point in TCR-signaling (upstream of IL-2 expression or solely by calcineurin activity), the switch decision for IL-2 expression is located. In general, different mechanisms for binary responses in gene expression have been discussed in eukaryotic cells [29]; [30]. Control steps in activation-induced IL-2 expression and relevant for this switch decision influenced by ionomycin or CsA could be (i) the IL-2 mRNA formation and its degradation, (ii) IL-2 promoter accessibility for the initiation of IL-2 mRNA transcription, (iii) cooperative and synergistic interaction between the transcription factors NFAT, NF-κB, and AP1 for IL-2 promoter binding, and (iv) the activation and translocation of the transcription factors NFAT and/or NF-κB, which are both known to be involved in IL-2 transcription as well as dependent on calcineurin activity.

So far, with two exceptions [31]; [32], the regulation of IL-2 production in primary T cells was studied in collections of cells and neither on sorted subpopulations nor on the level of single cells. Therefore, an on-off switch per cell could not be identified up to now. To overcome these limitations, we first used the IL-2 capture assay to separate IL-2 producing and non-producing cells after PMA/ionomycin stimulation. Second, we developed a method to stain isolated nuclei from human primary Th cells with NF-κB- and NFATc2-specific fluorescent antibodies and to measure them cytometrically.

As NFATc2 translocation into the nucleus is already an all-or-none decision per cell after partial activation of Th cells, IL-2 translation, mRNA formation and stability, and IL-2 promoter accessibility as the major switch decision step could be ruled out. The evidence point to active NFATc2 as regulated by the phosphatase activity of calcineurin to be the molecular switch for IL-2 expression per cell. First, we showed that in partially activated human memory cells, either all–or-none of the NFATc2 is dephosphorylated per cell. The population of IL-2 producing cells had mainly dephosphorylated NFATc2, while IL-2 non-producing cells contained mainly phosphorylated NFATc2. Second, it is known that CsA inhibits calcineurin activity by forming a CsA-Cyclophylin18-calcineurin-complex [33]. Graded concentrations of CsA lead to all-or-none NFATc2 but graded NF-κB nuclear translocation per cell. Third, titration of ionomycin induces a graded Ca^2+^ influx, which directly activates calcineurin activity [34]; [35] and results in an all-or-none expression of IL-2 per cell. Calcineurin is unique for its activation by Ca^2+^/calmodulin as well as its ability to dephosphorylate cellular NFAT [36]. In contrast to NFATc2, NF-κB is activated not only by ionomycin but need PMA in addition. Fourth, the NFAT-dependent gene IFN-γ [25] shows a binary expression pattern, while the NFAT-independent gene CD69 [26]; [27] shows a graded expression pattern in stimulated human memory Th cells.

Further support for NFATc2 dephosphorylation as the decision making step for binary IL-2 expression is coming from the hypotheses from Okamura et al. [24] . They suggested that cooperative dephosphorylation of 13 conserved serines by calcineurin promotes activation of an NFAT molecule by increasing the probability of an active conformation. In general, such a proposed molecular switch can become in addition a cellular switch if the responsible enzyme is working near substrate saturation [30]. Taken that the threshold for calcineurin activity is near the NFAT saturation, our mathematical modeling predicts that cooperativity of at least 7 of the 13 NFATc2 dephosphorylation sites would be sufficient to induce a binary IL-2 expression.

Our results are in contrast to the hypothesis suggested by Fiering et al. [19] and mathematically modeled by Pirone and Elston [20]. They proposed that binary reporter-gene expression is caused by stochastic DNA binding of NFAT and NF-κB as a randomly occurring process dependent on the transcription factor concentrations being above a certain threshold. This mechanism cannot account for the binary response of IL-2 expression after graded stimulation or graded inhibition of stimulated Th cells, as the all-or-none decision is already upstream of the nuclear translocation of NFATc2. Further experiments are needed to determine whether, among other possibilities, the limited availability of NFAT and/or NF-κB may be a reason why 39–62% of naïve Th cells and 8–30% of memory Th cells are not able to express IL-2 despite stimulation with optimal PMA/ionomycin concentrations.

**Figure 7:**
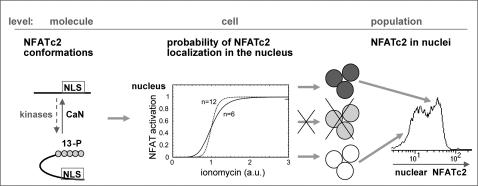
Schematic view of the NFATc2 switch. The NFATc2 switch can be regarded at the molecular, cellular and population levels as shown in the proposed model. First, at the molecular level, the dephosphorylation of 13 Serine residues of NFATc2 by calcineurin (CaN) leads to a conformational switch of an NFATc2 molecule from an inactive to an active conformation that exposes a nuclear localization sequence (NLS) and allows NFATc2 to enter the nucleus [24]. Second, at the cellular level, the nature of cooperativity of a minimum of 7 dephosphorylation sites (Hill coefficient n = 6) can account for a steep response curve and, consequently, a quantitative translocation of all the NFATc2 per cell by stimulation above a threshold with ionomycin. Third, at the population level, there are only cells with all-or-none NFATc2 in the nuclei.

The NFATc2 switch pictured in **Figure 7** combines the conformation model of NFAT molecules suggested by Okamura et al. [24] with the results in single cells presented here. TCR signaling translates TCR ligation into calcium response amplitude and duration [10]; [37]. Subsequently, calcineurin is activated and dephosphorylates NFATc2 at 13 conserved phospho-serine-residues, changing the conformation of NFATc2 and thereby unmasking an NFAT nuclear localization site (**Fig. 7**, left). Cooperativity of 7 or more of these dephosphorylation sites accounts for a steeply sigmoid response curve of NFAT activation after graded stimulation, causing a switch-like response of NFATc2 translocation per cell (**Fig. 7**, middle) and explains why only nuclei with either all or no NFATc2 were detected (**Fig. 7**, right). Therefore, TCR-dependent genes strictly depending on NFATc2 are expressed in an all-or-none fashion, which was shown for IL-2 in naïve and memory and for IFN-γ in memory Th cells. The stringent correlation between the frequencies of IL-2 and IFN-γ producing memory Th cells after graded stimulation with ionomycin or graded inhibition of stimulation by CsA confirms this view (**Supplementary Fig. S5**). The majority (90–92%) of IFN-γ producing memory Th cells co-express IL-2. In contrast, only 1/3 of the memory Th cells producing IL-2 have the potential to express IFN-γ after stimulation with either PMA/ionomycin or anti-CD3/CD28. Therefore, even if the NFATc2 switch is on and all NFATc2 is translocated into the nucleus in IL-2 producing cells, there appear to be other limitations preventing co-expression of IFN-γ in these cells.

What special advantages could the NFATc2-switch have in fine-tuning the adaptive immune response in vivo? First, this switch could help to drive a distinct fate decision of an individual T cell after activation. An all-or-none activation and translocation of NFATc2 can be important in the decision-making of a cell, preventing interstages of semi-activation, -anergy, and -differentiation into Th1 or Th2 or Treg which is rare. Indeed, the ability of Th cells to choose among launching a productive immune response, functional inactivation by anergy or apoptosis, or developing into regulatory T cells (only possible for naïve Th cells) depends upon the concentration and interplay of key transcriptional regulators, such as NFAT and AP-1 as well as on other transcriptional factors, cofactors and interaction partners which have yet to be identified [9; 38]–[40].

Second, the NFATc2-switch transforms the signal strength of antigen-specific T cell activation into the number of Th cells with a productive response. In general, the relative frequency of antigen-responding T and B cells determines the power of the adaptive immune response, depending upon localization of an antigen in a dose- and time-dependent manner [41]; [42]. The all-or-none decision of productive T cell activation by the NFATc2-switch induces that, at low antigen concentration activated cells are activated completely, and even at high antigen concentration, those that are not activated, are not activated at all. It is perhaps reasonable for the immune system to react to a higher antigen concentration with a higher number of activated Th cells actively participating in the immune response, while, in contrast, having all Th cells semi-activated could make it difficult to sharpen the outcome of an immune response [11]; [43].

In summary, this view and our data are in good agreement with the concept that signal strength drives T cells through various hierarchical thresholds for cell fate decision [43]. Moreover, the identified role of NFATc2 dephosphorylation is not just a threshold. It is rather an all-or-none decision to take the step to productive T cell activation. However, it has yet to be examined whether experimental results from in vivo studies are due to or can be explained by the NFATc2 switch. For example, it has to be clarified whether T cell deletion in vivo after weak antigenic stimulation is caused by the inability to switch-on NFATc2 in these Th cells, leaving them not “fit” and prone to disappear [43].

By translating the strength of antigenic T cell stimulation into the frequency of cytokine producing Th cells, the described NFATc2-switch is a general hub for productive adaptive immune response.

## Methods

### Human T cell isolation, stimulation, inhibition, and staining

Human Th cells from healthy volunteers were cultured in supplemented RPMI 1640. Phorbol 12-myristate 13-acetate (PMA; 10 ng/ml) and ionomycin (1 µg/ml) (Sigma Aldrich, Steinheim, Germany) were used to stimulate Th cells (5×10^6^/ml) or anti-CD3 (1 µg/ml) and anti-CD28 (4 µg/ml) (BD Biosciences, Heidelberg, Germany) immobilized to polystyrene beads (Polysciences, Eppelheim, Germany) for the times indicated.

Stock solutions of CsA (AWD, Dresden, Germany) in DMSO (Sigma, Deisenhofen, Germany) were used in a final concentration of 0.5% DMSO in each cell sample and were added 15 min before T cell stimulation.

CD4^+^ T cell isolation with a purity of>98% and intracellular cytokine staining were performed as previously described [44]. CD4^+^CD45RO^+^CD45RA^-^ memory Th cells were sorted by CD45RA depletion by either fluorescence activated or magnetic cell sorting with a purity of>98% and>85%, respectively. Antibodies used for T cell staining were APC-anti-IL-2, FITC-anti-CD45RA, FITC-anti-CD45RO (BD Pharmingen, San Diego, CA), PE-anti-CD69 and FITC-anti-IFN-γ (in house antibodies).

### IL-2 secretion assay

Human Th cells or memory Th cells (5×10^6^/ml) were preincubated with DMSO or CsA and stimulated with PMA and ionomycin for 2 h. IL-2 secretion assay was performed according to the manufactures instructions (Miltenyi Biotech, Bergisch Gladbach, Germany) with the following modifications: Stimulated Th cells were intensively washed with ice-cold PBS/BSA and incubated with an anti-CD45-anti-IL-2 bispecific antibody capture matrix (Miltenyi Biotech) for 3 min on ice. Cells were then transferred to warm complete RPMI medium at 37°C for 5 min with gentle mixing before IL-2 secretion was stopped with ice-cold PBS/BSA. The captured IL-2 was detected with APC-anti-IL-2 antibodies (Miltenyi Biotech) and IL-2 secreting and non-secreting cells were purified by cell sorting within 5 min for each sort.

### Preparation, staining and flow cytometry of nuclei

Memory Th cells (2×10^6^/ml) were stimulated for 2 h and nuclei were isolated by incubation with ice-cold buffer A (10 mM Hepes (pH 7.8), 8 mM MgCl_2_, 320 mM sucrose, 0.1% Triton-X 100, Protease Complete Inhibitors) for 15 min. Isolated nuclei were washed twice with buffer A (without Triton) and fixed in 3% formaldehyde. After permeabilization in 0.3% Triton-X 100 and staining with mouse FITC-anti-NFATc2 (BD PharMingen) or mouse FITC-anti-NF-κB p65 (Santa Cruz Biotechnology, Santa Cruz, CA), nuclei were counterstained with 1 µg/ml propidium iodide (PI, Molecular Probes, Eugene, OR) and analyzed by flow cytometry on a FACSCalibur (Becton Dickinson, San Jose, CA). Isolated nuclei were gated according to forward and side scatter and propidium iodide staining and excuded from doublets by pulse-processing (**Supplementary Fig. S3**).

### CMV-specific human T-cell lines

Human PBMC were stimulated with the cytomegalovirus peptide (TLGSDVEEDLTMTRNPQPF; 1 µg/ml) of the phosphoprotein 65 (pp65; JPT Peptide Technologies GmbH, Berlin, Germany) for 6 hours. IFN-γ Secretion Assay (Miltenyi Biotech, Bergisch Gladbach, Germany) was performed according to the manufactures instructions. IFN- γ secreting cells were isolated by magnetic cell sorting and expanded for 14 days with irradiated peptide-loaded autologous PBMC and rIL-2 (100 U/ml) ( Proleukin; Chiron Behring GmbH, Emeryville, CA). Autologous antigen-presenting cells (APC) were pulsed for 2 hours with either pp65 peptide (antigen-specific stimulation) or cytomegalovirus immediate-early protein 1 (IE-1) peptide (unspecific control). The expanded antigen-specific T cells (2×10^6^/ml) were incubated with the indicated amounts of CsA for 30 min. Peptide-loaded APC were then added to the antigen-specific T cells in the ratio of 1∶2. Brefeldin A (5 µg/ml) was added 2 hours later. After stimulation for 14 hours surface CD4 (PEcy7-anti-CD4, in house antibody) and intracellular IL-2 staining (APC-anti-IL-2, BD Pharmingen, San Diego, CA) was performed as previously described [44].

### IL-2 ELISPOT Analysis

The IL-2 ELISPOT assay was performed with isolated human memory Th cells (purity>98%) using the human IL-2 ELISPOT kit (BD Pharmingen, San Diego, CA) according to the instructions. Th cells in different dilutions (1×10^5^ to 3×10^1^/well) were stimulated over night with 10 ng/ml PMA and 1000 ng/ml ionomycin in the presence or absence of 4 nM CsA. The digitalized images were analyzed for spot number and diameter using the Bioreader-2000 (BioSys, Karben, Germany) plate reader with the software supplied by the manufacturer. Dilutions up to 300 cells per well allowed a good discrimination between the spots by the software. However, higher cell concentrations per well made the reduction of IL-2 producing cells in the CsA treated samples visible by eye.

### RT-PCR analysis

mRNA and cDNA preparation were performed with human Th cells (5×10^6^/ml) 2 h after stimulation using RNeasy- and Oligotex-Kit (Qiagen, Hilden, Germany). Reverse transcription was performed according to the manufacture's protocol using SuperSCRIPT II reverse transcriptase (Invitrogen, Karlsruhe, Germany). Primers for IL-2 cDNA amplification by Light Cycler were designed for an intron-exon junction (synthesized by TIB-MOLBIOL, Berlin, Germany): hIL-2 cDNA: forward, CACAGCTACAACTGGAGCATTTA, reverse AGAAATTCTACAATGGTTGCTGTC; h β2-Microglobulin cDNA forward TGGAGAGAGAATTGAAAAAGTGGAGC, reverse TTAAAAAGCAAGCAAGCAGAATTTGG. cDNA was amplified in duplicates using the Light Cycler FastStart DNA Master SYBR Green I Master Mix (Roche) and subjected to following conditions: 95°C for 10 min for one cycle; 95°C for 10 s, 58°C (IL-2 cDNA), 56°C (h β2-Microglobulin) for 10 s, and 1/25 bp amplicon for 72°C for 45 cycles. The data generated from each reaction were analyzed using Light Cycler software (Roche, Mannheim, Germany) and normalized to h β2-Microglobulin. To correlate the threshold (Ct) values from the respective cDNA amplification plots to copy number, a standard curve for each gene was generated.

### NFATc2 dephosphorylation

Purified human Th cells or memory Th cells (1×10^6^/ml) were stimulated for 1 h and 5 h with PMA/ionomycin, respectively. The latter were reversibly fixed in DSP (Pierce, Rockford, IL), intracellularly stained for IL-2, and separated by cell sorting for IL-2 producers and nonproducers. The fixation was reversed by heating the cell samples at 100°C for 15 min in Laemmli buffer. Immunoblotting (7.5% SDS-PAGE) was made using mouse anti-NFATc2 (Santa Cruz Biotechnology, Santa Cruz, CA) and anti-mouse-IgG peroxidase-conjugate (Sigma Aldrich Taufkirchen, Germany) or goat-anti mouse IgG_1_-IRDye 800 (LI-COR, Lincoln, Nebraska, USA). To quantify the NFATc2 bands of the sorted cell populations in Figure 4b, images were acquired with the Odyssey infrared imaging system (LI-COR Biosciences GmbH, Germany) and analyzed by the software program Odyssey 2.1. Total integrated fluorescence intensity of the phosphorylated and dephosphorylated NFATc2 bands were set to 100%.

### Mathematical modeling of cytokine expression

NFAT-dependent transcription of a cytokine gene was modelled as
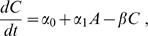
where C is the concentration of cytokine and A the concentration of active nuclear NFAT. The parameters α_0_, α_1_ and β denote the basal expression rate, the NFAT-induced expression rate constant, and the degradation rate constant, respectively. Active nuclear NFAT is taken to have a Hill-function dependent on the scaled ionomycin concentration I
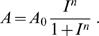
The Hill coefficient n is approximately proportional to how many serine residues are cooperatively involved in the regulation of the nuclear translocation of NFAT [28]. When *N* denotes the number of these residues, *n≈N*−1 for larger *N*. The steady state, obtained by setting *dC/dt* = 0 was taken as the cytokine expression level. To match the experimentally observed fluorescence intensities for IL-2, the following parameter values were chosen: α_0_/β = 5 (basal expression), α_1_ *A*_0_/β = 500 (NFAT-activated expression). To model random variations in basal transcription rate, calcineurin activity, and NFATc2 concentration, we simulated cytokine expression in 5000 cells, for which the parameters α_0_/β, α_1_ *A*_0_/β, and *I* were chosen from log-normal distributions with the mean values given above (and in Figure 6 for *I*). The standard deviation for each parameter was taken as 80% of the mean value.

## Supporting Information

Figure S1 Binary IL-2 expression in primary human Th cells using ELISPOT analysis. The number of IL-2 secreting peripheral memory Th cells (black bars) is reduced to 40% (SD = 8.5%) in the presence of 4 nM CsA (white bars). The average spot diameter did not differ significantly between the wells. These data confirm that IL-2 is expressed and secreted in a binary fashion. Representative results from two independent experiments.
(2.87 MB TIF)

Figure S2 Method for the detection of transcription factors in isolated single nuclei. The scheme illustrates the procedure for preparation, staining, and measurement of NFATc2 and NF-κB in isolated nuclei.
(1.63 MB TIF)

Figure S3 Gating strategy for the detection of stained nuclei. Single nuclei of primary human T cells were identified using forward and side scatter (a), propidium iodide staining (c) and exclusion from doublets by pulse-processing (b). A minimum of 10.000 nuclei, present in each of the three regions (R1- R3), were gated for further analysis of the transcription factor.
(2.50 MB TIF)

Figure S4 Specific detection of NFATc2 and NF-κB in isolated nuclei by flow cytometry. The specificity of NFATc2 staining (left) was confirmed using nuclei from stimulated human Th cells pre-treated with a specific inhibitor of calcineurin (10 nM CsA) and an agent to reduce the level of cytosolic Ca2+ (50 mM Ca2+ chelator EGTA). The specificity of NF-κB (p65) staining (right) in nuclei from PMA/ionomycin-stimulated human Th cells was confirmed by NF-κB staining in nuclei of cells stimulated with TNF-alpha an alternative and weaker inducer of NF-κB in T cells.
(0.93 MB TIF)

Figure S5 Co-staining of IL-2 and IFN-γ. IL-2 and IFN-γ were detected by intracellular staining 5 hours after PMA/ionomycin stimulation. The frequencies of IL-2 producing, IFN-γ producing, as well as IL-2 and IFN-γ co-producing cells were determined (upper part) at different ionomycin concentrations (constant PMA 10 ng/ml) and different concentrations of cyclosporine A during PMA/ionomycin stimulation (lower part). One representative experiment out of two is shown.
(2.25 MB TIF)
