# Supplementary figures and images for: Digital NFATc2 Activation per Cell Transforms Graded T Cell Receptor Activation into an All-or-None IL-2 Expression
Source: PLoS One. 2007 Sep 26;2(9):e935. doi: 10.1371/journal.pone.0000935 (PMC1978524; doi:10.1371/journal.pone.0000935)

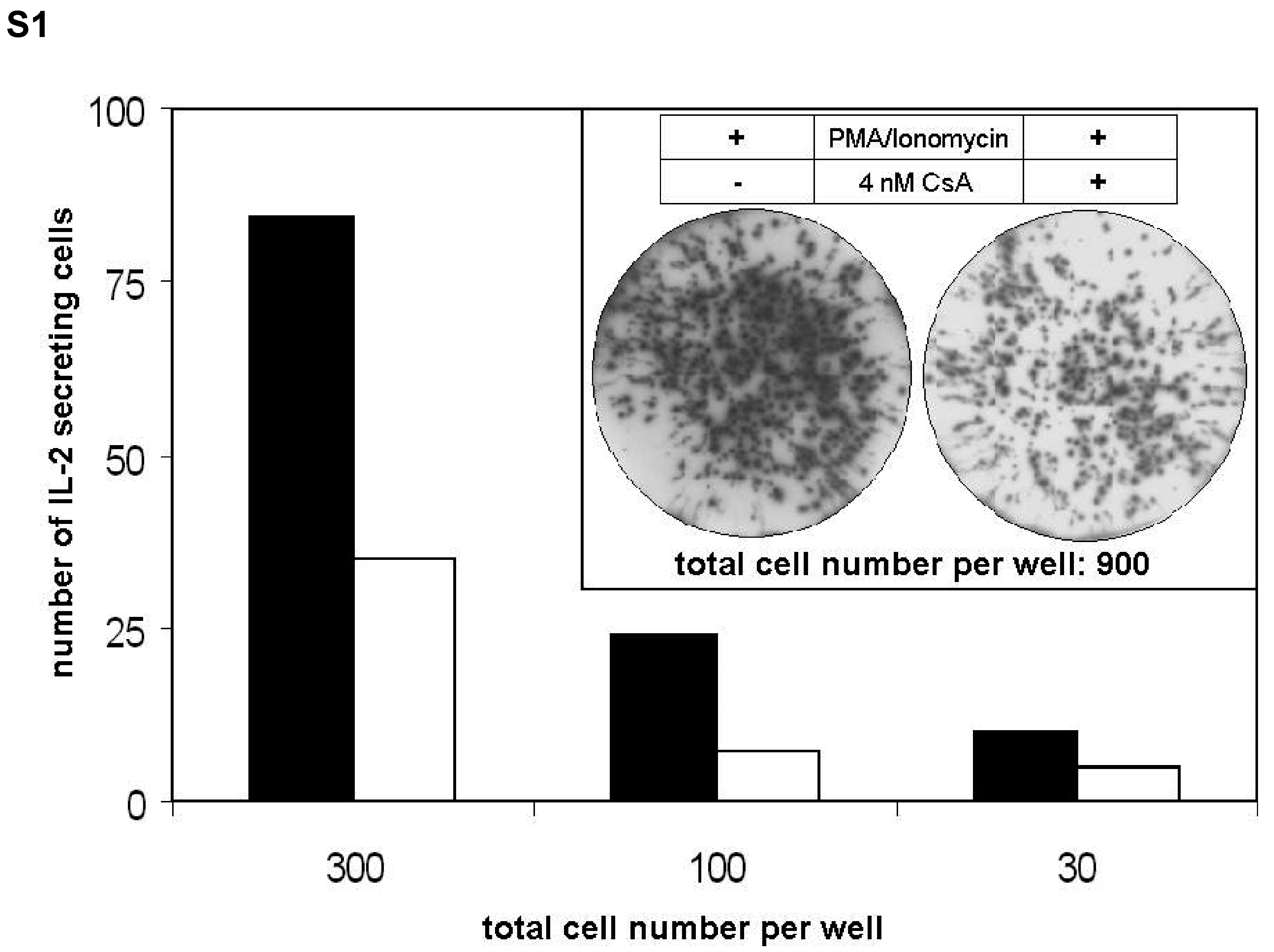

Supplement: Figure S1 — Binary IL-2 expression in primary human Th cells using ELISPOT analysis. The number of IL-2 secreting peripheral memory Th cells (black bars) is reduced to 40% (SD = 8.5%) in the presence of 4 nM CsA (white bars). The average spot diameter did not differ significantly between the wells. These data confirm that IL-2 is expressed and secreted in a binary fashion. Representative results from two independent experiments. (2.87 MB TIF) [file pone.0000935.s001.tif]

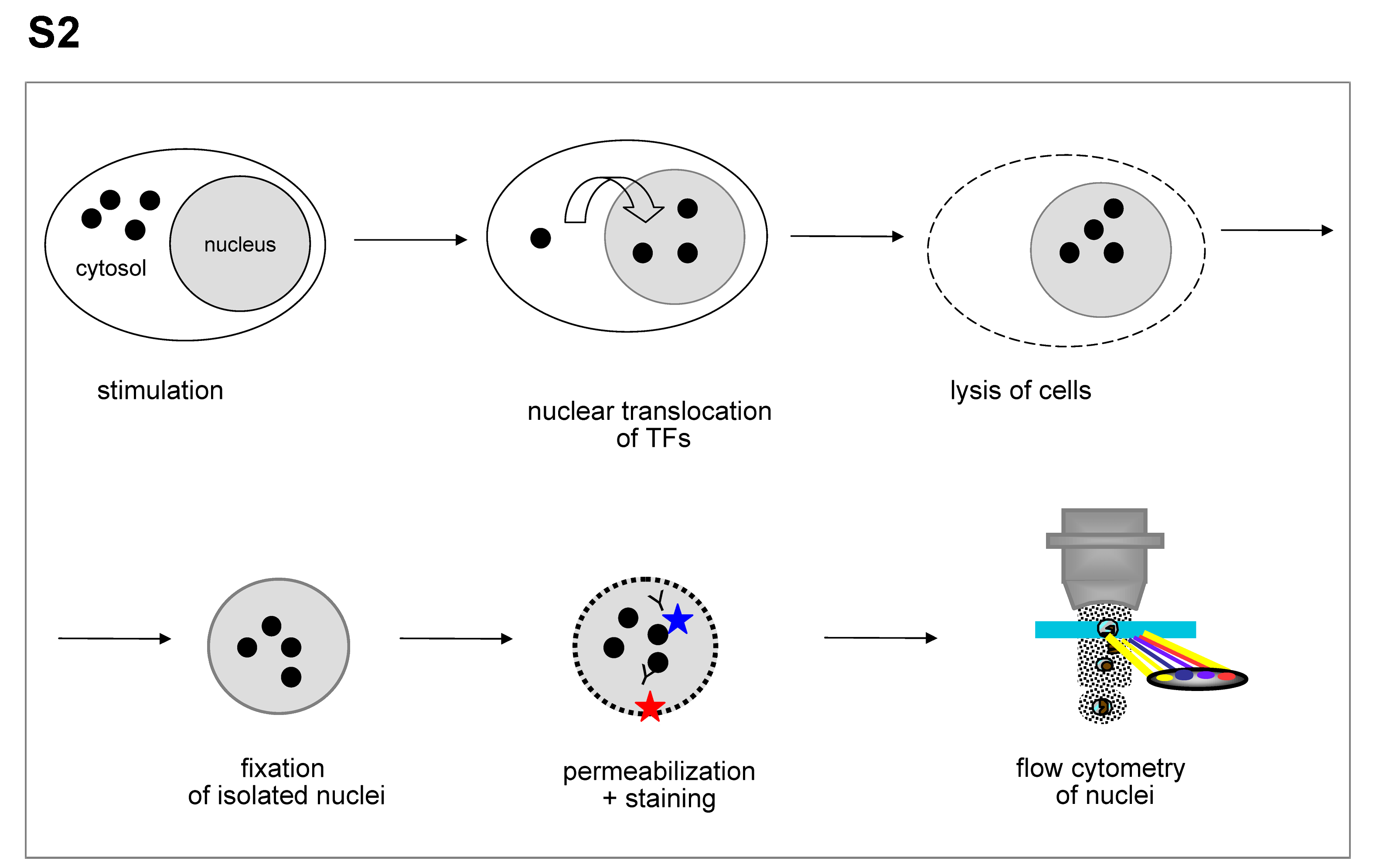

Supplement: Figure S2 — Method for the detection of transcription factors in isolated single nuclei. The scheme illustrates the procedure for preparation, staining, and measurement of NFATc2 and NF-κB in isolated nuclei. (1.63 MB TIF) [file pone.0000935.s002.tif]

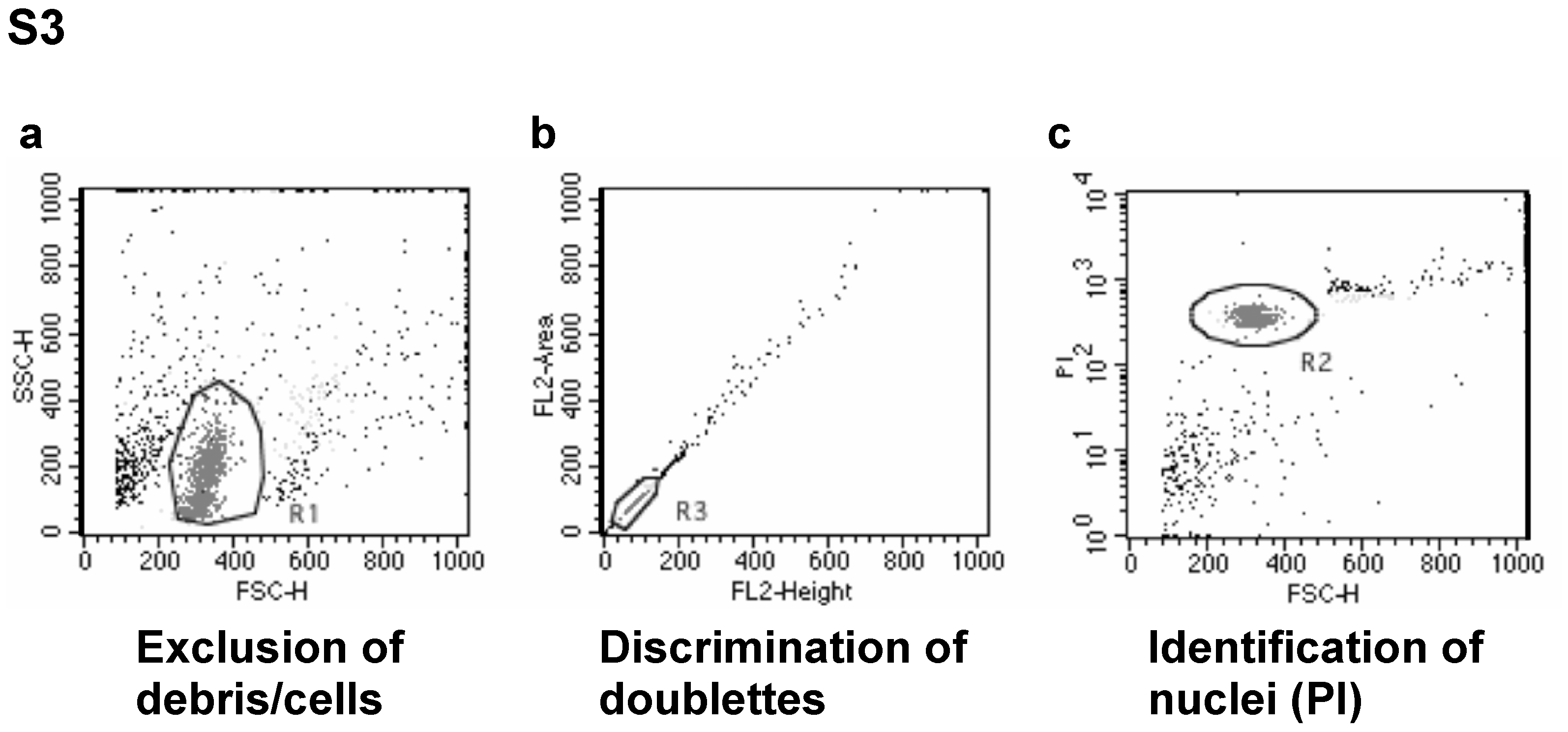

Supplement: Figure S3 — Gating strategy for the detection of stained nuclei. Single nuclei of primary human T cells were identified using forward and side scatter (a), propidium iodide staining (c) and exclusion from doublets by pulse-processing (b). A minimum of 10.000 nuclei, present in each of the three regions (R1- R3), were gated for further analysis of the transcription factor. (2.50 MB TIF) [file pone.0000935.s003.tif]

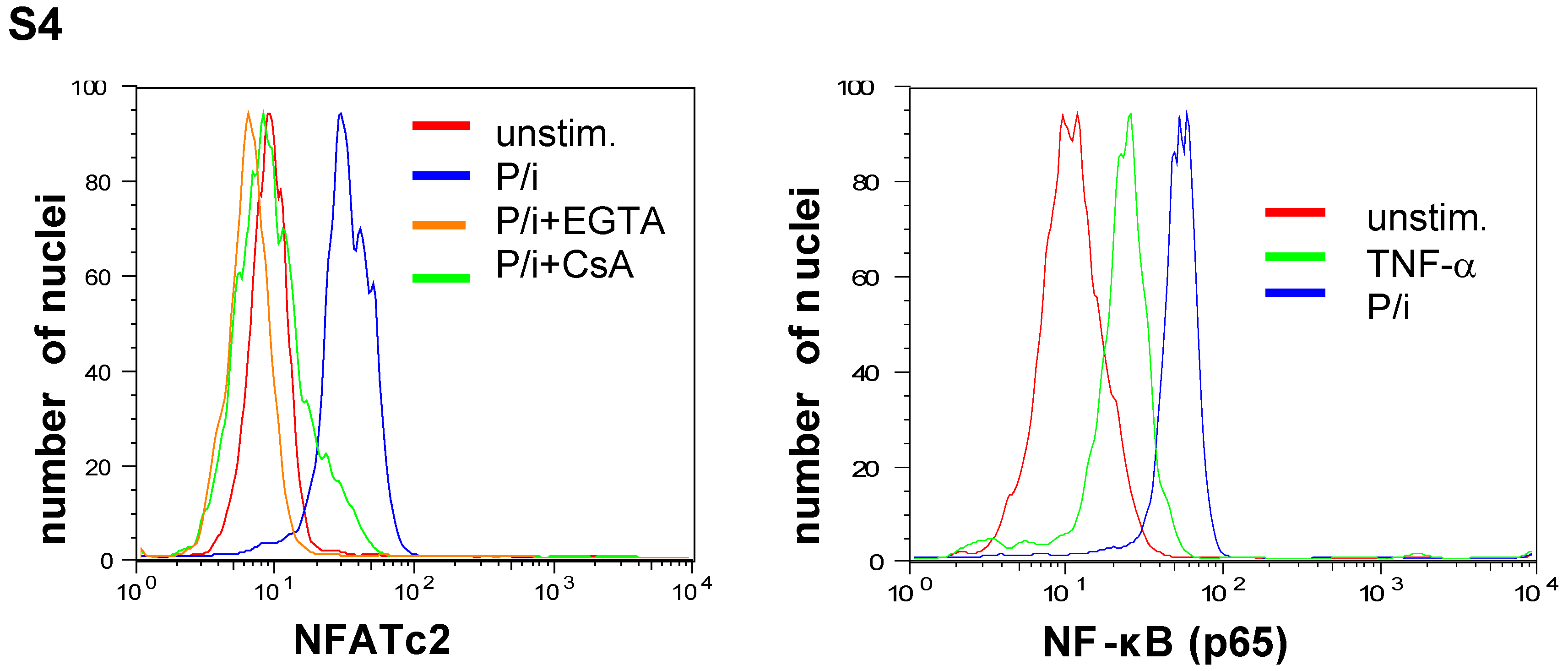

Supplement: Figure S4 — Specific detection of NFATc2 and NF-κB in isolated nuclei by flow cytometry. The specificity of NFATc2 staining (left) was confirmed using nuclei from stimulated human Th cells pre-treated with a specific inhibitor of calcineurin (10 nM CsA) and an agent to reduce the level of cytosolic Ca2+ (50 mM Ca2+ chelator EGTA). The specificity of NF-κB (p65) staining (right) in nuclei from PMA/ionomycin-stimulated human Th cells was confirmed by NF-κB staining in nuclei of cells stimulated with TNF-alpha an alternative and weaker inducer of NF-κB in T cells. (0.93 MB TIF) [file pone.0000935.s004.tif]

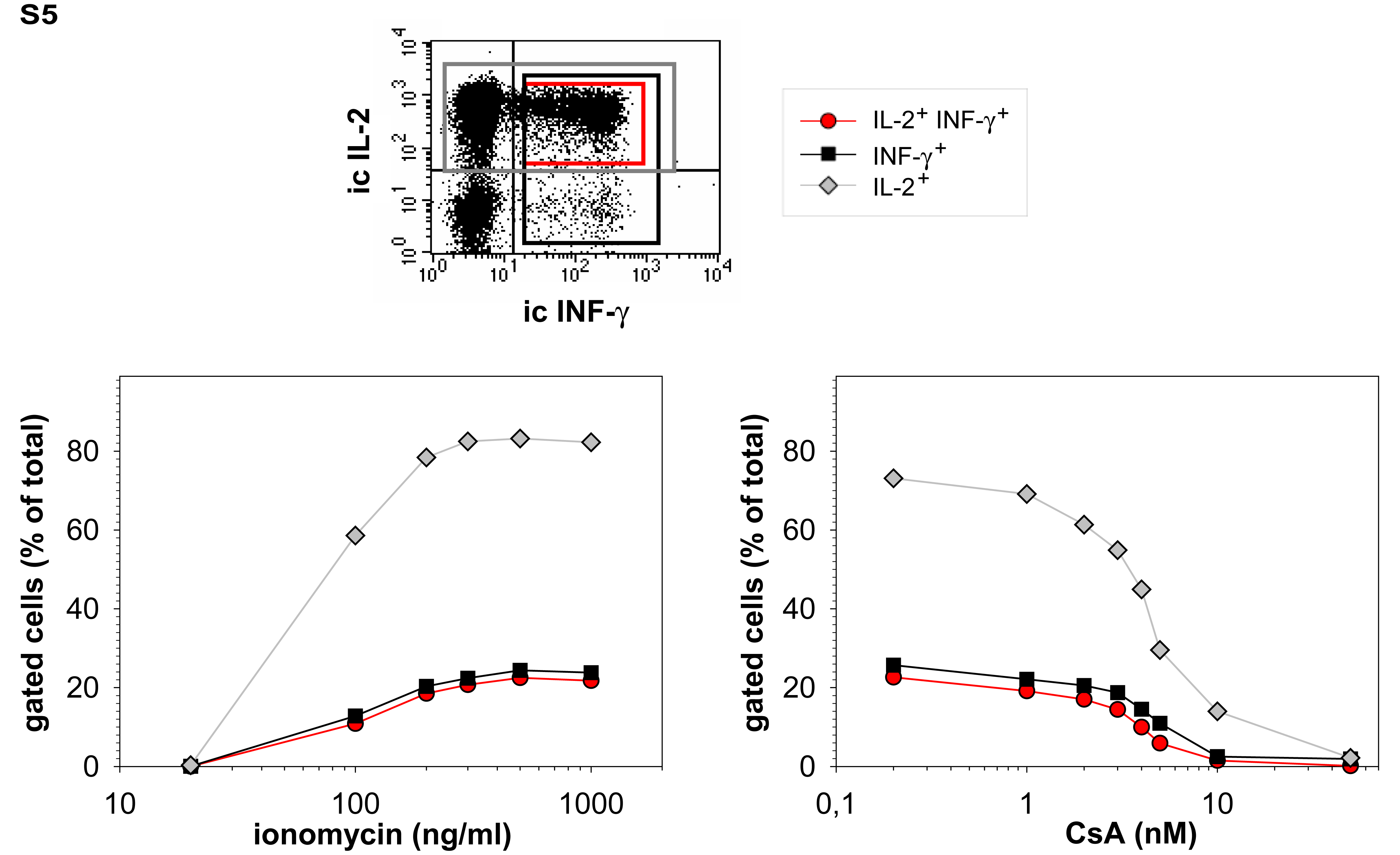

Supplement: Figure S5 — Co-staining of IL-2 and IFN-γ. IL-2 and IFN-γ were detected by intracellular staining 5 hours after PMA/ionomycin stimulation. The frequencies of IL-2 producing, IFN-γ producing, as well as IL-2 and IFN-γ co-producing cells were determined (upper part) at different ionomycin concentrations (constant PMA 10 ng/ml) and different concentrations of cyclosporine A during PMA/ionomycin stimulation (lower part). One representative experiment out of two is shown. (2.25 MB TIF) [file pone.0000935.s005.tif]
